# Perceptive Recommendation Robot: Enhancing Receptivity of Product Suggestions Based on Customers’ Nonverbal Cues

**DOI:** 10.3390/biomimetics9070404

**Published:** 2024-07-02

**Authors:** Masaya Iwasaki, Akiko Yamazaki, Keiichi Yamazaki, Yuji Miyazaki, Tatsuyuki Kawamura, Hideyuki Nakanishi

**Affiliations:** 1 Graduate School of Engineering Science, Osaka University, Osaka 560-8531, Japan; iwasaki@irl.sys.es.osaka-u.ac.jp; 2 School of Media Science, Tokyo University of Technology, Tokyo 192-0982, Japan; ayamazaki@stf.teu.ac.jp; 3 Graduate School of Humanities and Social Sciences, Saitama University, Saitama 338-8570, Japan; yamakei@mail.saitama-u.ac.jp; 4 Graduate School of Interdisciplinary Information Studies, The University of Tokyo, Tokyo 113-0033, Japan; yujimppm@g.ecc.u-tokyo.ac.jp; 5 Independent Researcher, Kyoto, Japan; k@drgn.style; 6 Faculty of Informatics, Kindai University, Osaka 577-0813, Japan

**Keywords:** HRI, field experiment, sales robot, service robot

## Abstract

Service robots that coexist with humans in everyday life have become more common, and they have provided customer service in physical shops around the world in recent years. However, their potential in effective sales strategies has not been fully realized due to their low social presence. This study aims to clarify what kind of robot behavior enhances the social presence of service robots and how it affects human–robot interaction and purchasing behavior. We conducted two experiments with a sales robot, Pepper, at a retail shop in Kyoto. In Experiment 1, we showed that the robot’s social presence increased and that customers looked at the robot longer when the robot understood human gaze information and was capable of shared attention. In Experiment 2, we showed that the probability of customers picking up products increased when the robot suggested products based on the humans’ degree of attention from gaze and posture information. These results indicate that the robot’s ability to understand and make utterances about a customer’s orientation and attention effectively enhances human–robot communication and purchasing motivation.

## 1. Introduction

Robots that provide customer service in physical stores are being utilized to coexist with humans in everyday life. The use of service robots instead of human service workers is expected to reduce labor costs by providing services such as multi-language support and more efficient ways to introduce products to local and foreign customers. However, it is still unclear how to effectively use service robots for sales and product promotion, and their potential has not been fully realized. One reason for this is that the social presence of robots is low, and their statements and suggestions are often ignored. In recent years, many studies have been conducted using customer service robots, but most of them have been limited to providing recommendation information about the products [1,2]. Although other sales strategies such as offering samples [3] and encouraging customers to pick up products [4,5,6] have been shown to be effective for sales, such robots’ customer service behaviors are rarely utilized. There are several definitions of social presence, but in this study, we defined it as “the degree to which humans feel that robots can interact socially with others,” and we attempted to solve the above problem by enhancing the social presence of robots. Therefore, we set up the following two research questions:

**RQ1:** How can we increase the social presence of robots so that they are not ignored?

**RQ2:** How can we make people accept the robot’s suggestions after listening to them?

We conducted the following two experiments to address each of these research questions.

**Experiment 1: Shared attention for enhancing social presence:** We investigate what kinds of behaviors can enhance the social presence of robots so that humans do not ignore them. It has been shown in previous studies that expressing an understanding of human states is effective in enhancing the social presence of robots [7]. Moreover, interacting with objects in human space gives the impression of sharing space, which is effective in enhancing the social presence of robots [8]. Therefore, the purpose of this experiment is to enhance the social presence of the sales robot by enabling shared attention with the customers. Shared attention occurs when both parties share an interest in an object [9,10]. Moreover, human gaze is said to have an expressive function of conveying intention and emotion among non-verbal information [11], and there are studies that show that robots can infer human intention from gaze information and initiate collaborative behavior [12]. In this study, the robot senses customers’ gaze information and expresses that it understands it, giving the customers the impression that the sales robot shares the space with them and enabling the robot to share attention with the customers. Therefore, in this experiment, we examine whether the robot can enhance its social presence by showing that it understands a customer’s gaze information.

**Experiment 2: Posture recognition for improving the acceptance of suggestions:** We investigate what kinds of behaviors can prompt customers to take products according to the robot’s suggestions in order to enable them to accept the robot’s suggestions. We think that the robot needs to propose products with awareness of the customer’s gaze in order to make them pick up the product. We considered that the customer’s attention level to the product is related to the posture, and we can estimate the attention level to the product by detecting the customer’s posture. In related studies, human preferences were investigated for a robot’s eye gaze behavior during human-to-robot handovers [13]. Another study demonstrated that the gaze control approach that controls the robot’s head movement according to the human’s gaze is perceived as more natural and humanlike in human–robot interaction [14]. Furthermore, a study proposed motion control of a walking assistance robot based on human state observation using wearable sensors [15]. In this study, we aimed to enhance the acceptability of the service robot’s recommendation for promoting purchase behavior in the actual store environment by focusing on the customer’s gaze and posture information. Additionally, based on previous studies showing that the possibility of purchasing is high after taking the product [4,5,6], we utilized the suggestion to persuade the customer to physically pick up the product. Therefore, we aim to investigate whether we can increase the possibility of accepting the robot’s suggestion by utilizing personalized information such as the customer’s posture. This study aims to examine whether we can increase the probability of customers accepting the robot’s suggestions by using the customers’ posture information to make suggestions in the actual store environment.

## 2. Related Works

### 2.1. Service Robots

Service robots are used in various places, and many studies have been conducted. For example, they are used in museums [16,17,18,19,20], educational settings [21,22], hotels and airports [23,24,25]. There has been a study that used robots as sales staff in a shopping mall and provided coupons to increase sales [26]. However, in this experiment, the robot did not directly introduce the products to the customers. Some studies robotize the products themselves and encourage customers to pick them up while moving [27,28]. However, this method is limited to the products and situations to which it can be applied, and it is not easy to apply to all products in the store. Another study showed that the recommendations accompanied by the robot pseudo-eating behavior can achieve the same recommendation capability as other existing recommendation methods [29]. However, it is unclear what kind of pseudo-experience should be expressed in the recommendation of non-food products. Therefore, this study examines an effective method that does not limit the target products, using a customer service robot that can directly approach customers.

### 2.2. Shared Attention (Joint Attention)

Joint attention is achieved by sharing interest in the object of attention while both parties direct their attention to the same object [9,10]. Joint attention is considered an important social skill and is suitable for modeling robots [30,31,32]. Therefore, many studies have been conducted on human–robot joint attention, and it has been shown that joint attention is effective in making robots competitive [33] or attractive [34,35] to humans. However, few experiments have been conducted in actual field environments. In this study, we investigated whether the same effect would occur for customers in a real store.

### 2.3. Social Presence

In a previous study, social presence was defined as “the sense of being together with another and mental models of other intelligence that help us simulate other minds” [36], and it is a critical indicator in human–robot interaction. Moreover, it was found that social presence enhanced fun [24], satisfaction [37], trust [38], the user’s persuasive ability and motivation to interact with the robot [39,40]. To increase this social presence, it is effective to use joint attention [41] and reactions to others’ behavior [7].

## 3. Method

**Figure 1:**
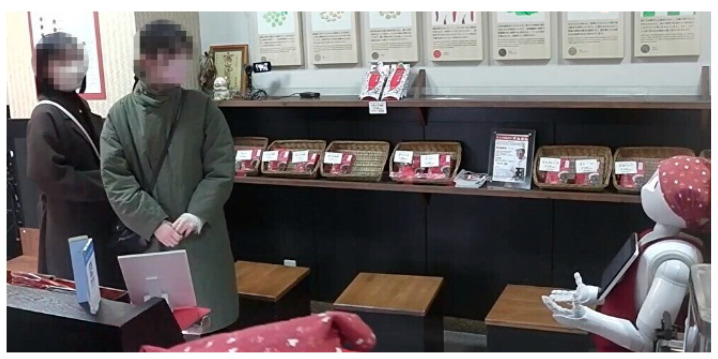
One of the scenes in the experiments.

Figure 1 shows a field experiment conducted at a specialty shop for shichimi (a Japanese spice blend) called “Dintora” in a shopping district in Kyoto. This experiment was carried out with the approval of the research ethics committee of Osaka University.

**Figure 2:**
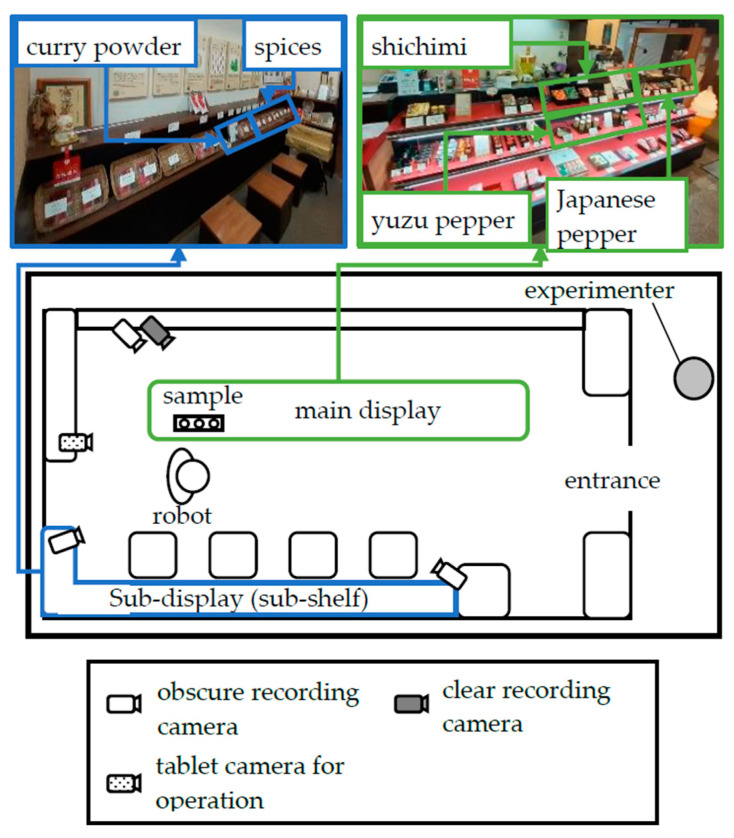
Layout of the shop.

**Figure 3:**
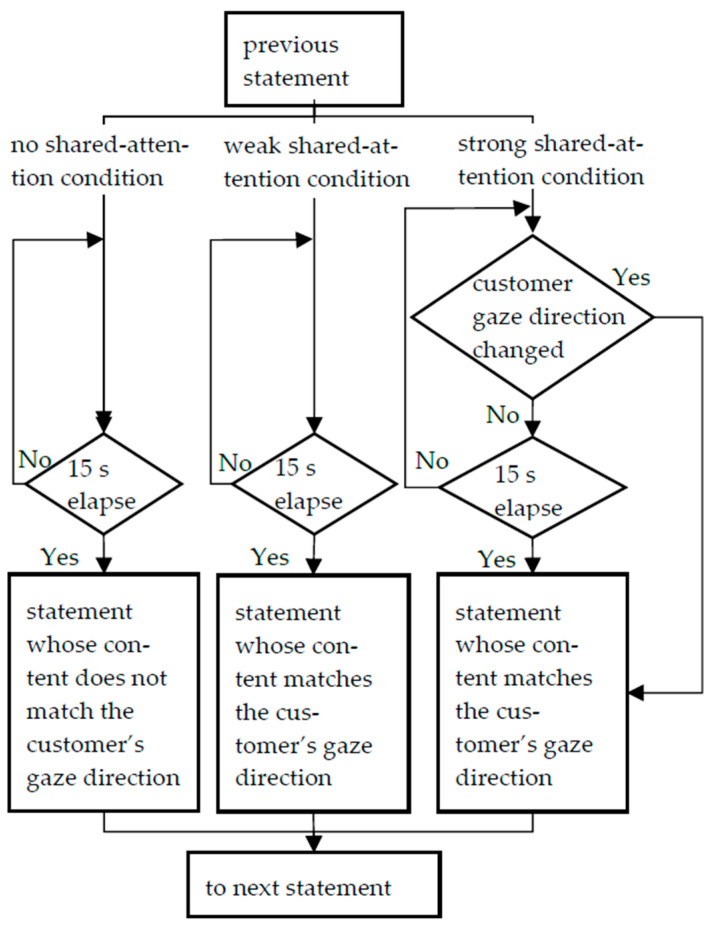
Robot’s behavior transitions in each condition.

Figure 2 is a top view of the shop’s interior space where the experiment was conducted, and there are shelves on the left and right, as shown in Figure 3. For recording purposes, we installed three obscure recording cameras, one clear recording camera and one webcam that captured customers’ movements. The clear recording camera focused on the robot’s side and recorded only customers who gave consent. In addition, we placed a tablet behind the robot so that the experimenter could observe the situation in the shop during the experiment. The tablet was connected to the experimenter via video call.

In this experiment, we adopted the humanoid robot Pepper as a customer service robot because of its moderate size and high safety. The experimenter stood where he/she could see the whole store and observed the situation in the shop with both the operation camera and the naked eye. The experimenter adopted the Wizard of Oz method and operated the robot with a smartphone.

## 4. Experiment 1: Shared Attention for Enhancing Social Presence

The first research question, RQ1, explores how to improve robots’ social presence so that humans do not ignore them. In this chapter, we explain Experiment 1, corresponding to this research question. In this experiment, we examined whether the robot could enhance its social presence by showing that it understands the customer’s gaze information.

### 4.1. Hypotheses

Human gaze is said to be nonverbal information with an expressive function to convey intentions and emotions [11]. In addition, there is research suggesting that interacting with objects in human space gives the impression of sharing space, which is effective in enhancing the social presence of robots [8].

Based on these previous studies, we have considered that expressing the robot’s understanding of the customer’s gaze information gives the customer the impression that the sales robot shares the space, thus enhancing the robot’s social presence. We conducted an experiment in a real store to investigate how the content and timing of robot utterances affect the enabling of shared attention between the customer and the robot and the social presence of the robot. Based on the above, we proposed the following two hypotheses.

**Hypothesis** **1.** *The robot’s statements regarding the customer’s gaze improve its social presence.*

**Hypothesis** **2.** *The robot’s statements when the customer’s gaze direction changes improve the robot’s social presence.*

### 4.2. Conditions

To test the above hypotheses, we designed two factors and three conditions as follows: 

**Factor 1:** The match between the robot’s utterance content and the customer’s gaze information. 

**Factor 2:** The match between the robot’s utterance and the timing of the customer’s gaze changes. 

**No-shared-attention condition:** The robot’s gaze is not shared with the customer’s gaze. Every 15 s, the robot describes a product on the opposite shelf from the one the customer is looking at while facing forward. 

**Weak-shared-attention condition:** The robot mentions the customer’s gaze direction and enables the customer and the robot to share attention. The robot’s utterance interval is the same as in the “no-shared-attention condition.” That is, the robot describes a product on the same shelf as the customer while looking in the same direction as the customer. 

**Strong-shared-attention condition:** Basically, the robot’s utterance content and interval are the same as in the “weak-shared-attention condition,” except that when the customer changes the gaze direction from one shelf to another, the robot immediately describes the product on the shelf the customer is looking at, instead of making periodic utterances. The robot’s utterance frequency and length was controlled to be the same in every condition. Table 1 and Table 2 show the robot’s utterances in each condition. The robot’s utterances were in Japanese only, and the utterance order was predetermined. Figure 1 shows the behavior transitions in each condition. During the experiment, the experimenter observed the customer’s gaze direction from the tablet’s operation camera, determined which of the three directions (main display, sub-display or robot) the customer was facing and operated the robot accordingly.

### 4.3. System

The experimental setup was the same as described in Chapter 3. Twelve buttons were created on the smartphone for the robot to speak about the product information on the main display and the sub-display. The experimenter used these buttons to control the robot remotely according to the situation.

### 4.4. Results and Discussion

**Figure 4:**
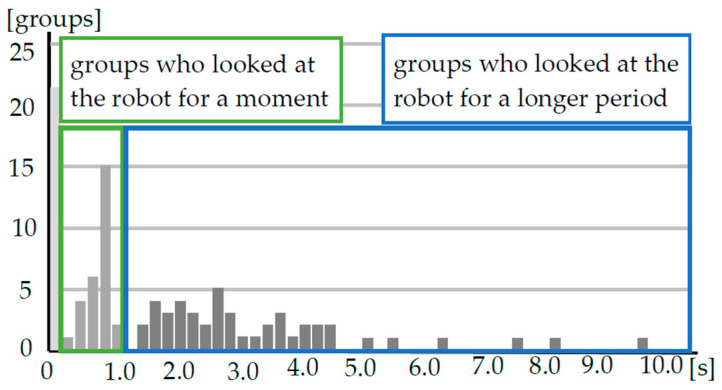
Relationship between time spent looking at the robot and total number of customers.

**Figure 5:**
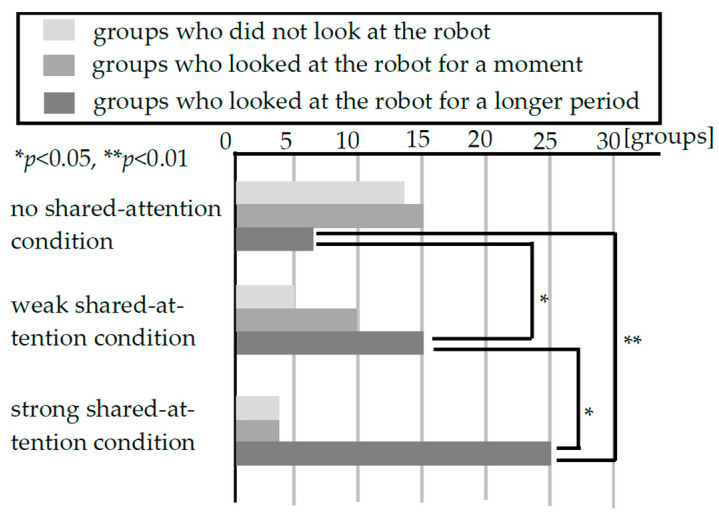
Customer responses in each condition.

The experiment was conducted over six days in 2020 with 95 pairs of customers. Previous studies have shown that the longer a customer makes eye contact with the robot, the higher the robot’s social presence [42,43]. Therefore, we used eye contact with the robot as an indicator of the robot’s social presence. We measured the time that the person closest to the robot in each customer group looked at the robot from video recordings of the experiment. The average robot gaze times for each condition were 0.91 s for the no-shared-attention condition, 1.89 s for the weak-shared-attention condition and 2.65 s for the strong-shared-attention condition. However, some customers looked at the robot for reasons other than the robot’s high social presence. For example, some customers were surprised by the robot’s sudden statement and looked at the robot to verify the source of the voice. To exclude such customers, we divided those who looked at the robot into two groups: “a group that looked at the robot briefly (less than 1 s)” and “a group that looked at the robot longer (more than 1 s).” We set the threshold at 1.0 s because it is estimated that it takes about 1 s for a customer to turn toward the robot to identify the source of the voice. Figure 4 shows the relationship between the time spent looking at the robot and the total number of customers. The results indicate that there was a gap between the distributions of the groups at 1.0 s. Therefore, we categorized all customers into three groups: those who did not look at the robot, those who looked at the robot for less than 1 s and those who looked at the robot for more than 1 s; we evaluated these groups separately. Figure 5 shows the results concerning the customers’ responses in each condition. When we applied Fisher’s exact test to these analyses, we found significant differences among the three groups (*p* < 0.05). Therefore, we performed multiple comparisons between groups. Figure 5 shows that there are significant differences between the no-shared-attention condition and the weak-shared-attention condition (supporting hypothesis 1) (*p* < 0.05), between the weak-shared-attention condition and the strong-shared-attention condition (supporting hypothesis 2) (*p* < 0.05) and between the no-shared-attention condition and the strong-shared-attention condition (*p* < 0.01) only in the group that looked at the robot longer. This suggests that when the robot uttered information about the customer’s gaze direction and timed its utterance to the customer’s gaze change, the number of groups that looked at the robot longer increased.

In the following, we analyze typical, detailed human–robot interactions in each condition using ethnomethodology and conversation analysis. Conversation analysis, which emerged from the influence of ethnomethodology, is used in analyzing the sequential structure of human interactions [44,45]. Goodwin revealed that both embodied actions and the material world/environment are resources in human interactions, in addition to verbal actions [46]. In the transcripts, we transcribed the human–robot interactions to match the word order and meanings between the original language and English.

The transcription conventions are as follows: 

(0.0): numbers in parentheses indicate the time of a pause. 

(.): indicates very short silence.

[: indicates the onset of overlap.

=: indicates no “gap” between the two lines.

:: indicates sound stretching, with the number of colons indicating the relative length of the stretching.

(( )) describes what people are doing, who they are looking at and so on.

(h): indicates laughter during speech.

* Gaze direction of a customer is described as follows:

To R: gaze towards the robot; to S: gaze towards the sub display; to F: gaze towards the food sample.

Abbreviations—SR: robot; Cx: customer (number); SC: shop clerk; S: side display.

#### 4.4.1. Content of the Robot’s Statements

To examine the interaction in more detail, we show typical fragments for the weak-shared-attention condition and no-shared-attention condition (Fragment 1 and Fragment 2). In the weak-shared-attention condition, in Fragment 1, the customer saw the product (line 7) and the robot mentioned it (line 8). This means that the robot’s remark was matched to the direction of the customer’s orientation, enabling shared attention. In the no-shared-attention condition corresponding to Fragment 2, the robot’s statement was not matched to the customer’s orientation (line 1); therefore, shared attention between the robot and the customer was not enabled. The difference between Fragment 1 and Fragment 2 is whether the robot’s statement about the direction of the customer’s gaze caused the content of the statement to match the orientation of the person. This is the main reason for the difference of customer responses in these two conditions, which supports hypothesis 1.

**Fragment 1.** Weak-shared-attention condition

07 C1: ((looking at the main display))

08 SR: What you [are seeing now is Yuzu Pepper=

09 C1: [((to R for 0.6 s))

10 =amazing That’s the correct answer

11 C1: ((to R for 1.6 s))

**Fragment 2.** No-shared-attention condition

01 SR: Shichimi soft [serve is delicious

02 C2: [Wow [(.) I was surprised::

03 C2: [((to R for 0.8 s))

04  ((C1 does not show any reaction thereafter))

#### 4.4.2. Timing of Robot’s Statements Regarding Customer’s Gaze

**Figure 6:**
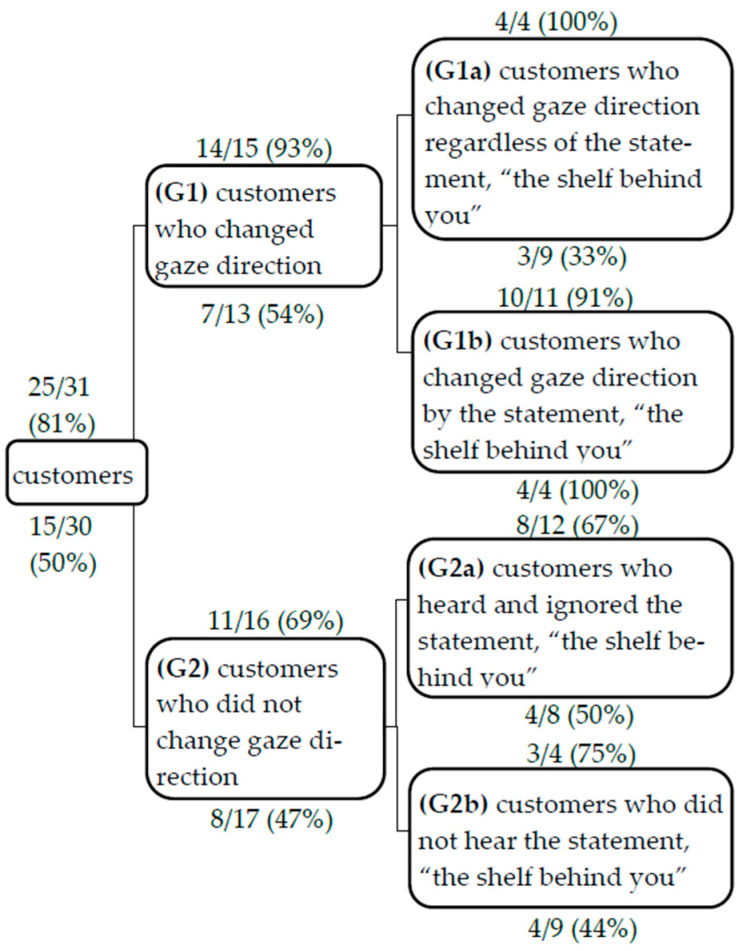
Customer behaviors in the strong-/weak-shared-attention conditions. The numbers above and below each box are the percentage of groups who looked at the robot for a long time/number of applicable groups. The number above is for the strong-shared-attention condition, and the number below is for the weak shared condition.

**Figure 7:**
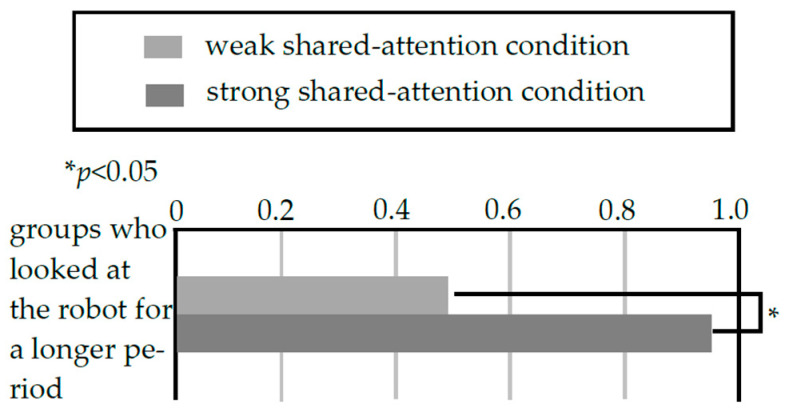
Post hoc analysis result (responses of groups who changed their gaze from one shelf to another).

In this experiment, it was necessary for the robot to speak in accordance with the timing of the customer’s gaze change. Therefore, the key phrase “the shelf behind you” was introduced for both the strong-shared-attention condition and weak-shared-attention condition to make it easier for customers to change their gaze. In this way, all groups in the strong-shared-attention condition and weak-shared-attention condition could be classified into two groups: one in which the customers’ gaze changed (G1) and one in which it did not (G2). We further classified G1 into two groups: G1a, the group whose gaze changed depending on the key phrase, and G1b, the group whose gaze changed regardless of the key phrase. Finally, G2 was classified into two subcategories: G2a, those who heard the key phrase and ignored it, and G2b, those who did not hear the key phrase. Figure 6 shows the number of groups that exhibited the above characteristics in the strong-shared-attention condition and weak-shared-attention condition. To examine the influence of the robot’s statements when customers changed their gaze direction, it is necessary to eliminate the influence of the groups that did not change their gaze direction. Therefore, we conducted a post-hoc analysis of the groups that changed their gaze direction in the weak- and strong-shared-attention conditions. As shown in Figure 7, there was a significant difference (*p* < 0.05) between the two conditions because of Fisher’s exact test in the number of groups of customers who looked at the robot for a long time among customers who changed their gaze direction. 

Thus, the robot’s statements about the direction in which the customer was facing increased the robot’s social presence, which supported hypothesis 2. To clarify the reason for the difference between these two conditions, we present typical fragments of the strong-shared-attention condition and weak-shared-attention condition (Fragment 3 and Fragment 4). In Fragment 3, from the strong-shared-attention condition, when the customer’s gaze changed to a product in the sub-display (line 13), the robot made a statement that matched the customer’s gaze information (lines 14–17). The customer then attempted to find the specific product (line 18) and turned his body toward a food sample. Therefore, the customer engaged in the activity that the robot suggested in its statements. In contrast, in the weak-shared-attention condition, in Fragment 4, when one customer’s gaze turned to the products on the sub-display and back to the main display (lines 3–9), the robot did not make a remark. A few seconds later, the robot made a remark referring to the sub-display, but the customer did not respond again. Thus, the timing of the robot’s utterance was one of the determinants that allowed shared attention between the robot and the customer. 

In both the strong- and weak-shared-attention conditions, many groups changed their gaze with the phrase “the shelf behind you” (Figure 6). We re-examined Fragment 3 under the strong-shared-attention condition in response to these results. C3, standing in front of the main shelf, turned around and looked at the shelf behind them (lines 12–13). Here, the robot’s statement implicitly induced the customer to pay attention to another spice in the store, and C3 reacted by looking toward the shelf. One of the reasons the robot’s statement guided the customer was the characteristic of the sentence structure, that is, its particularity. When the robot said, “On the shelf behind you, there are spices as well,” this invited the customer (standing in front of the main display) to turn and find unexpected spices in the traditional Japanese shop. It was an implicit invitation. The robot’s statement constrained the customer’s following action in the invitation–acceptance/rejection adjacency pair of human–robot interaction [47]. 

The varying proportion of groups who looked at the robot for a long time between the two conditions within G1a (shown as percentages in Figure 6) was a primary factor in showing the difference between the strong and weak conditions in Figure 5. Because there was no constraint on adjacent pair of phrases here, only the timing of the robot’s statement was considered an important determinant. Therefore, timing the robot’s statement regarding the customer’s gaze was vital in enabling shared attention between the customers and the robot.

**Fragment 3.** Strong-shared-attention condition

11 C3: Oh((looking at products in the main display while walking))

12 SR: On behind the shelf [there are spices, too

(Translation: On the shelf behind you, there are spices as well)

13 C3: [((to R))][((to S)) 

14 SR: [What you

15 : are looking [curry powder, I recommend

16 C3: [((going to S)) 

17 SR: in particular

(Translation: I recommend you the curry powder you are looking at right now, in particular)

18 C3: Curry powder? ((to R))

19 SR: Do you want to try some?

20 C3: ((to R, then turning his body to F, then to R))

21 SR: Here, you can [try

22 C3: [((to F))

(Translation: You can try it here)

**Fragment 4.** Weak-shared-attention condition

01 SR: Shichimi on the shelf is deli[cious

02 C4: [((to R))

03 : ((turning her gaze to the products in the sub-display))

04 (2.0) 

05 C4: ((pointing and looking at the poster

near SD))

06 C4: ((looks at the poster))

07 (8.0)

08 C4: [((turns towards the main display))

09 C5: [((turns towards the main display))

10 (3.0)

11 SR: On behind the shelf, there are spices, too

    (Translation: There are spices on the shelf behind you as well.)

## 5. Experiment 2: Posture Recognition for Improving the Acceptance of Suggestions

The second research question, RQ2, explores how to make humans accept the robot’s suggestions after listening to them. In this chapter, we explain Experiment 2, corresponding to this research question. In this experiment, we examine whether the robot can enhance the acceptability of its suggestions by proposing products using the customers’ degree of attention based on their posture information.

### 5.1. Hypotheses

In Experiment 1, based on the research suggesting that interacting with objects in human space gives the impression of sharing space, which is effective in enhancing the social presence of robots [8], we attempted to enhance the social presence of the robot by giving the impression of sharing the space by having the robot speak based on the gaze information. In Experiment 2, in order to strengthen this impression more, we investigated whether customers were more likely to pick up the products suggested by a robot based on the estimated interest level from the customers’ posture information. As indicators of the interest level, we consider two factors: whether the customers were looking at the products and whether they were leaning forward when looking at the products. Based on this, we set up the following two hypotheses:

**Hypothesis** **3.** *By prompting customers to pick up products while they are looking at them, the number of customers who pick up the products will increase.*

**Hypothesis** **4.** *By prompting customers to pick up products while they are leaning forward to look at them, the number of customers who pick up the products will increase.*

### 5.2. Conditions

To verify the hypotheses, the following conditions were set: 

**No-gaze-awareness condition:** The robot would say “Please try picking up the product” when the distance between the customer and the robot was less than 2 m, as the robot was located approximately 4 m from the store entrance. 

**Low-gaze-awareness condition:** When the customer looked at a product after entering the store, the robot said, “Please try picking up the product that you are looking at now, the [product name].”

**High-gaze-awareness condition:** When the customer was leaning forward to look at a product after entering the store, the robot would say, “Please try picking up the product that you are looking at now, the [product name]”.

The robot’s statements were prepared in Japanese and English, and each condition was performed only once. Some customers left the store without meeting the conditions for the robot’s statement.

### 5.3. Experimental Setting

The experimental setup was the same as described in Chapter 3. The system used in this experiment is described below.

#### 5.3.1. Customer Posture Detection

**Figure 8:**
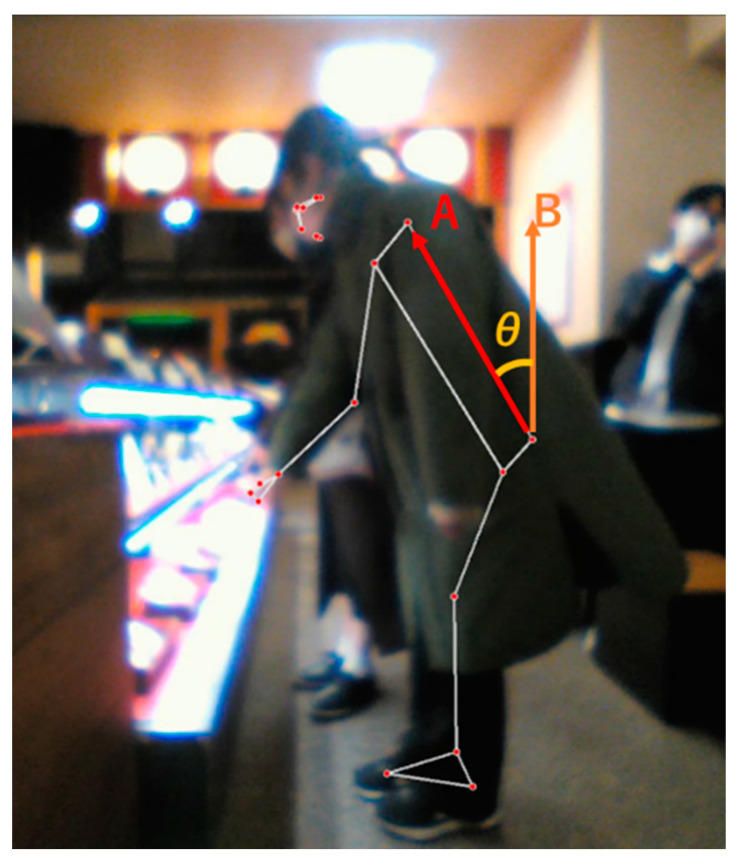
Example of customer posture detection using MediaPipe. (A: vector from the waist to the shoulder; B: upward vertical vector; θ: angle formed by A and B).

In this experiment, we set the condition that the robot would make a statement if the customer leaned forward while looking at the product. To avoid ambiguity in judging whether the customer was in the specified posture, we considered using a quantitative value to make the judgment. Therefore, MediaPipe was used in this study to determine the customer’s posture. As shown in Figure 8, the coordinates of the customer’s hands, shoulders, waist and feet were obtained from the video captured by the webcam using MediaPipe, and vectors were generated from each coordinate; the customer’s posture was determined from the angle between the vectors.

#### 5.3.2. Posture Criteria

In this experiment, it was necessary to establish a criterion for determining whether the customer assumed a peering posture. To this end, we examined the angle (θ) formed between the vector from the waist to the shoulder (A in Figure 8) and an upward vertical vector (B in Figure 8) in ten customers who assumed a peering posture before experimenting. Our analysis revealed that a customer was deemed to have assumed a peering posture if the angle θ was 37° or more, which was the average value of all ten customers. In this study’s high-gaze-awareness condition, this average value was used as the threshold value, and only those who clearly peered into the display were treated as people who assumed a peering posture.

### 5.4. Results and Discussion

**Figure 9:**
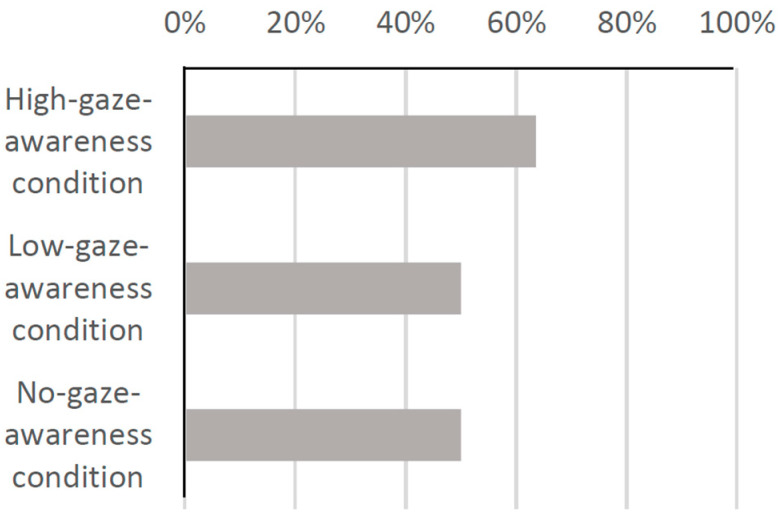
Percentages of groups who picked up the product.

The experiment was conducted over five days in 2022, and 50 groups of customers visited. To exclude groups that left the shop quickly, groups that stayed in the shop for less than 30 s were excluded from the analysis, and 41 groups were used as data for the analysis. Among the 41 groups, 14 were under the no-gaze-awareness condition, 16 were under the low-gaze-awareness condition and 11 were under the high-gaze-awareness condition. Figure 9 shows the percentage of groups who picked up products when they visited the shop under each condition. From this figure, it can be seen that 64% of the groups picked up products in the high-gaze-awareness condition, while 50% of the groups did so in the low-gaze-awareness and no-gaze-awareness conditions. This suggests that serving customers under the high-gaze-awareness condition is more effective in encouraging them to pick up a product than the other two conditions. However, no statistical analysis was performed in this experiment due to the small sample size. In a previous study, a robot suggested that visitors pick up products in a certain store, similar to our experiment [27]. It was reported that the product pickup rate for the proposed method in this study was 281 out of 8189 passersby (3.4%). The pickup rate in our system was 64%, which is relatively high compared to previous studies, but the situation may be slightly different. In our study, we examined the product pickup rate among those who entered the store. Therefore, in this study, it would be more consistent with our situation to consider 281 out of 901 (31.2%) of those who stopped in front of the system as the product pickup rate. Comparing this result to our results, we believe that our system was effective enough.

From the perspective of ethnomethodology and conversation analysis, we analyzed the interaction between customers and the robot, examining how the robot’s verbal cues when customers showed interest in a product affected their behavior.

The following are typical examples of customers’ responses for each condition.

**Fragment 5.** No-gaze-awareness condition

01 C1 ((Move to the center of the store while looking at the main display))

02 SR Please take the [product in your hands!

03 C1          [((Look at Sub display))

04         (8.0)

05 C1  ((Turn around and look at Main display))

**Fragment 6.** Low-gaze-awareness condition

01 C2 ((Look at “Container”))

02 SR Please pick up [“Container” you are [looking at right now.=

03 C2 [((look at SR for 0.8s))

04 C3 [((look at SR for 0.7s))

05 C3 =huhuhuhu

06 C2 Thank you.

07 C2 ((Move to Entrance))

**Fragment 7.** High-gaze-awareness condition

01 C4 ((Look at “Ichimi”))

02        (5.0)

03 C4 ((Approaching Main display and look into “Ichimi”))

04 SR Please pick up [“Ichimi” you are looking at right now.

05 C4          [((look at SR for 3.6s))

06 SR ((Return her gaze to “Ichimi”))

07 SR ((Pick up “Ichimi”))

An example of a group that responded with no gaze awareness is shown in Fragment 5. When this group reached the shop’s center, the robot made a statement (lines 01–03). The customer did not react to the statement, instead shifting their gaze towards a different shelf during the robot’s statement (line 04). In Fragment 6, an example of a group that responded to the low-gaze-awareness condition. When this group was looking at one of the products, a container, the robot made a statement (lines 01–02). The customer then laughed and thanked the robot (lines 03–06) but subsequently shifted their gaze away from the container towards a different item (line 07). An example of a group that responded in the high-gaze-awareness condition is shown in Fragment 7. After the group had been in the shop for some time and had been looking at the products, they approached the product shelf and peered in when the robot made a statement (lines 01–05). The customers then looked at the robot, returned their gaze to a product and picked it up (lines 06–07). 

In Fragment 5, the robot’s statement was completely ignored, and the customer did not pick up any products. On the other hand, in Fragment 6, the customer looked at the robot for a short time (0.8 s) but still did not pick up any products. Moreover, as shown in Figure 9, there was no significant difference in the number of groups who picked up products between the no-gaze-awareness and the low-gaze-awareness conditions. Therefore, it is possible that the robot’s suggestions were not being accepted under either condition, which may refute hypothesis 3. The reason for this could be that most of the customers were always looking at products in the shop after they entered, and the robot’s statement came when they were already looking at a product, regardless of the condition. As a result, there was no difference in the customers’ attention to the products between the no-gaze-awareness and the low-gaze-awareness conditions, which may have led to no difference between the two conditions.

**Figure 10:**
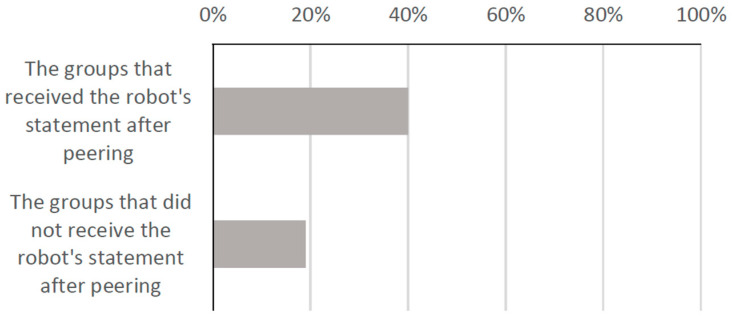
Percentage of groups who picked up the product after peering.

To examine the effect of the robot’s statement, we also investigated the customers’ behavior when peering into the display regardless of receiving the statement from the robot. The results showed that 40% of the groups picked up the product among the groups that received the robot’s statement after peering, and 19% of the groups picked up the product among the groups that did not receive the robot’s statement after peering as shown in Figure 10. This graph indicates that the robot’s statements had a significant impact on the customers’ behavior.

In Experiment 1, to exclude those who only briefly looked at the robot to confirm the source of the voice, participants who looked at the robot for 1.0 s or more were defined as groups who looked at the robot for a longer period, and the social presence of the robot was investigated. Furthermore, in Experiment 2, as shown in Fragment 7, unlike the examples shown in Fragments 5 and 6, the robot did not say anything while the customers were standing and looking at the product. Instead, the robot spoke when the customers approached the shelf and examined it. As a result, the customers looked at the robot for 3.6 s, which was longer than in the other two conditions. After that, they listened to the robot’s speech and finally picked up the product, which may support hypothesis 4. According to previous research [38], the longer the eye contact between the customer and the robot, the higher the level of social presence, which indicates the extent to which social interaction with another person is perceived.

**Figure 11:**
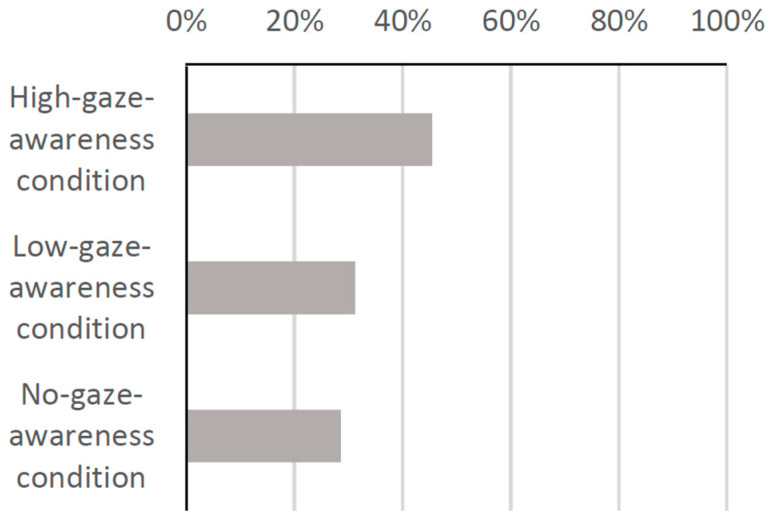
Percentage of groups who looked at the robot for a long period.

Therefore, we used the same method in Experiment 2 and again conducted a post hoc analysis. We investigated whether the social presence of the robot was enhanced in the high-gaze-awareness condition by examining the proportion of the groups who looked at the robot for a more extended period in each condition of Experiment 2. As a result, as shown in Figure 11, the proportion in the high-gaze-awareness condition was higher than in the other two conditions, indicating that the social presence of the robot was enhanced in the high-gaze-awareness condition compared to the other two conditions.

**Figure 12:**
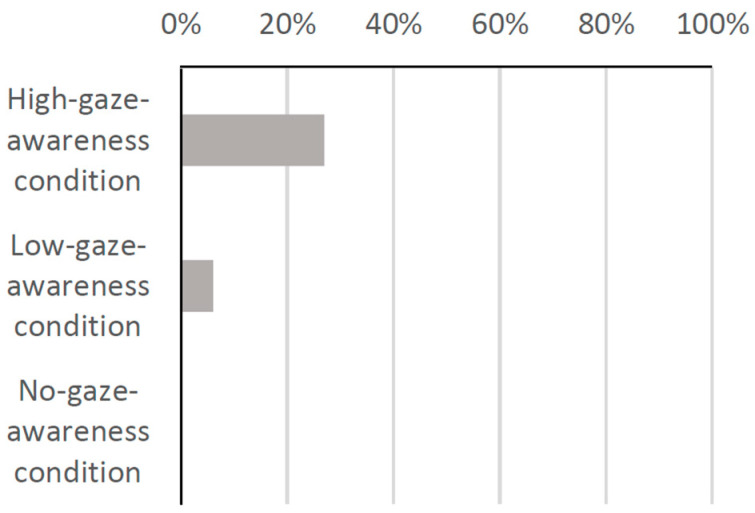
Percentage of groups who picked up the product after looking at the robot.

Based on these findings, we investigated the number of groups that picked up the product after looking at the robot. In the high-gaze-awareness condition, 27% of groups picked up the product after looking at the robot, compared to 6% in the low-gaze-awareness condition and 0% in the no-gaze-awareness condition as shown in Figure 12. This graph demonstrates that a higher percentage of groups picked up the product after receiving suggestions from the robot in the high-gaze-awareness condition than in the other two conditions.

Therefore, it can be considered that by making a statement when the customers were in the posture of peering at the products, the robot’s social presence was strengthened, leading the customers to accept the robot’s statement and pick up the product.

## 6. Conclusions

In this study, we aimed to examine what kind of robot behavior enhances the social presence of service robots and how it affects human–robot interaction and purchasing behavior. We set up the following two research questions: (RQ1) How can we increase the social presence of robots so that they are not ignored? (RQ2) How can we make people accept the robot’s suggestions after listening to them? To address each of these research questions, we conducted two experiments in a spice store with a sales robot that could sense human gaze and posture information and utter accordingly. As a result, we revealed that shared attention is enabled and the robot’s social presence increases when the robot understands human gaze information and makes a statement about it. Moreover, it was found that when the robot was strongly aware of the customers’ attention, 64% of the groups of customers picked up the product, which was 14% higher than when the robot had low or no such awareness. Therefore, the robot’s social presence is enhanced and humans increase their acceptance of the robot’s suggestions when the robot makes utterances according to the customers’ degree of attention based on human gaze and posture information. 

This experiment was conducted in a shop selling a specific product, shichimi. It can be expected that the robot’s social presence will be enhanced and humans will increase their acceptance of the robot’s suggestions when the robot makes utterances according to the customers’ degree of attention, even in situations involving different products. However, it remains to be verified whether the same method is effective in different situations. In addition, we used Pepper as the customer service robot in this study. However, there is research showing that robots with different appearances have different attributions of robot representations [48]. Whether the method used in this experiment is effective for robots of different sizes or appearances needs to be examined in the future.

The results of this study suggest that a robot’s capacity for awareness to understand and make statements about human attention and intention effectively enhances human–robot communication and purchase motivation, which contributes to the field of human–robot interaction.

## Figures and Tables

**Table 1:** The robot’s statements (no-shared-attention condition).

When a Customer Looks at the Main Display (Main Shelf)	When a Customer Looks at the Sub Display (Sub Shelf)
Shichimi soft serve is delicious.	Shichimi soft serve is delicious.
We have a wide variety of spices.	Shichimi is our specialty and I recommend it.
Curry powder is especially good.	Japanese Pepper is very tangy and stimulating
Cinnamon goes great with coffee and tea.	Yuzu Kosho goes well with meat.
I also recommend mustard and Wasabi.	Yuzu Shichimi is also very tasty.
Sesame seeds are made with the finest ingredients.	Ichimi is blended perfectly and exquisite.

**Table 2:** The robot’s statements (weak-/strong-shared-attention conditions).

When a Customer Looks at the Main Display (Main Shelf)	When a Customer Looks at the Sub Display (Sub Shelf)
The Shichimi on that shelf is delicious.	There are spices on that shelf.
On the shelf behind you, there are spices as well	Shichimi on the shelf behind you is the specialty.
What you are looking at now is Yuzu Kosho.	I recommend you the curry powder you are looking at right now in particular.
Japanese Pepper on that shelf is very popular!	Pepper near you is also very good.
There are also products at the back of the store on the shelf behind you.	The product on the shelf behind you is popular.
The Ichimi you are looking at now is excellent.	The Cinnamon you are looking at now goes great with coffee.

## Data Availability

All related data is included in the main text.
